# Metastatic ductal adenocarcinoma of the breast presenting with pericardial effusion—Challenges in the diagnosis of breast cancer

**DOI:** 10.1002/ccr3.2497

**Published:** 2019-10-24

**Authors:** Michael Chahin, Karan Seegobin, Satish Maharaj, Karishma Ramsubeik

**Affiliations:** ^1^ Division of Internal Medicine Department of Medicine University of Florida College of Medicine Jacksonville FL USA; ^2^ Division of Rheumatology and Clinical Immunology Department of Medicine University of Florida College of Medicine Jacksonville FL USA

**Keywords:** breast cancer, false negative, pericardial effusion

## Abstract

Breast cancer is a common entity that can be difficult to diagnose. This case demonstrates the limitations of breast cancer diagnostics. Particularly, how the available imaging techniques and even biopsy can potentially miss a malignancy. It exemplifies the role immunohistochemistry staining plays in the diagnosis of cancers of unclear origin.

## Graphical abstract

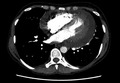

## 1 BACKGROUND

A 49‐year‐old female with a history of lupus presented with shortness of breath. Previous evaluation of breast masses with breast mammography, MRI, and biopsy had benign findings. Pericardiocentesis revealed adenocarcinoma. Excisional biopsy of axillary and supraclavicular lymph node yielded triple‐negative ductal breast adenocarcinoma. She was treated with abraxane and atezolizumab.

Breast cancer is a common entity but can be difficult to diagnose despite modern advances. Occult breast cancer is a rare entity, up to approximately 1% of breast cancers, with an age of peak incidence of approximately 55 years.^1^ These patients are at risk for presenting with symptoms of metastatic disease at an advanced stage. The presence of malignant pericardial effusions, and axillary or supraclavicular lymphadenopathy, should heighten suspicion for an underlying breast malignancy even in the presence of benign breast imaging.

## 2 CASE PRESENTATION

A 49‐year‐old female with a past medical history of hypothyroidism and systemic lupus erythematosus (SLE), manifested by positive ANA, arthritis, history of serositis, and history of hemolytic anemia presented with shortness of breath for 1 day after a recent viral illness 3 days prior to admission. She had been on hydroxychloroquine 100 mg twice daily and levothyroxine 125 mcg once daily. On examination, her blood pressure was 121/85 mm Hg, pulse 76 bpm, respiratory rate 25 bpm, and sPO2 99%. S1 and S2 were soft, and there were no murmurs. Air entry was equal bilaterally, and no crackles or wheezes were heard. Abdomen was soft and nontender, without masses. There was 2 cm nontender, mobile lymphadenopathy in the left supraclavicular and left axillary region, and 2 cm nodular lumps palpable on both breasts.

Her complete blood count showed white cell count of 6 × 10^3^ µL, hemoglobin 12 g/dL, and platelet 179 × 10^3^ µL. Her renal function test showed sodium 125 mmol/L, potassium 4 mmol/L, calcium 8 mg/dL, and creatinine 0.9 mg/dL. Other laboratory tests showed ANA 1:320 (+) with speckled pattern, anti‐DS DNA (−), anti‐RNP (−), anti‐Smith (−), anti‐SS‐A positive, anti‐SS‐B positive, C3 119 (90‐180 mg/dL), and C4 28 (10‐40 mg/dL).

**Figure 1:**
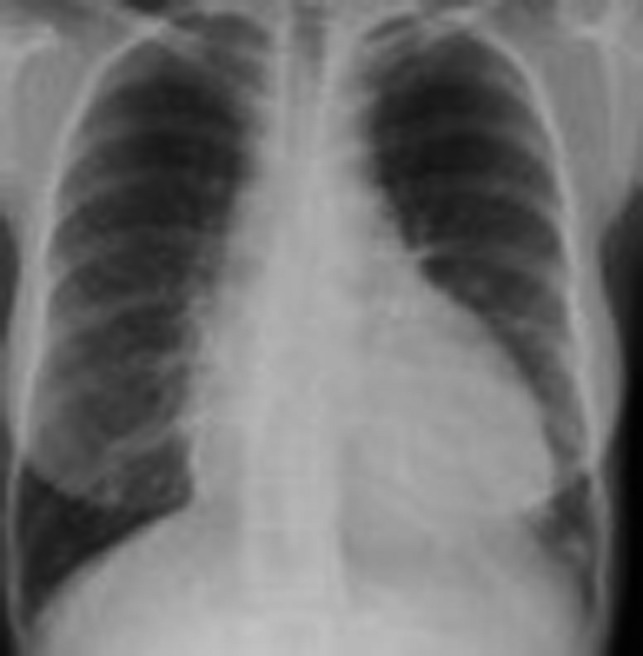
CXR revealed a large cardiac silhouette

**Figure 2:**
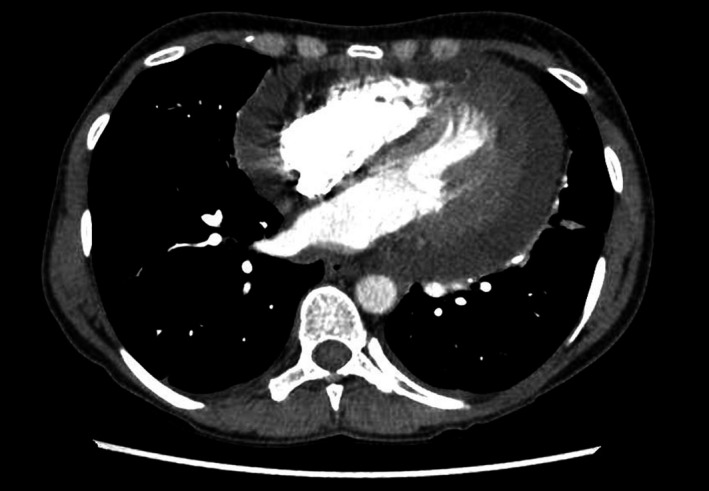
CT chest revealed a large pericardial effusion

Her chest X‐ray showed an enlarged cardiac silhouette (Figure 1). CT chest revealed a large pericardial effusion (Figure 2).

Echocardiography showed ejection fraction of 55%‐60% with pericardial effusion and thickened pericardium.

She underwent pericardiocentesis yielding 700 mL of bloody effusion. Biopsy of the pericardium showed nonspecific inflammation.

The cytology of the pericardial fluid was positive for metastatic adenocarcinoma and was CK7, and GATA‐3 positive.

**Figure 3:**
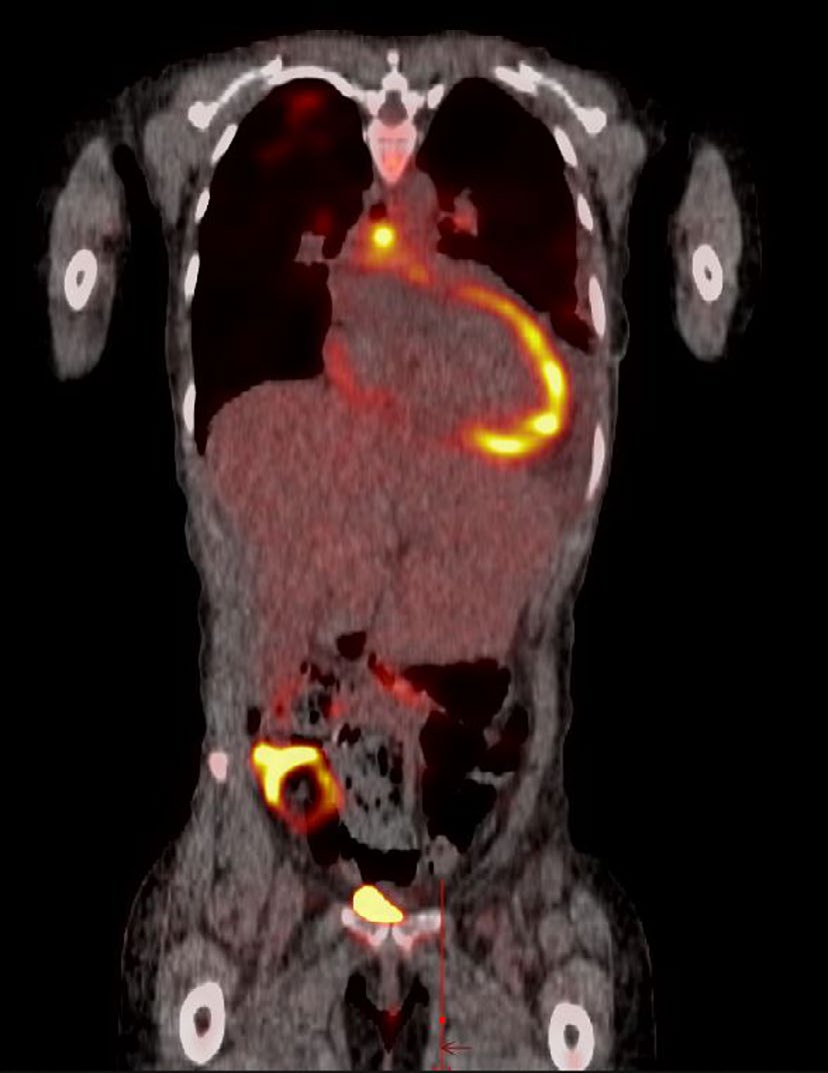
PET scan revealed metabolically active left axillary, bilateral supraclavicular, anterior mediastinal, subcarinal, and left internal mammary lymphadenopathy

Her CT chest abdomen and pelvis were significant for left axillary, bilateral supraclavicular, and anterior mediastinal lymphadenopathy up to 2 cm. Her PET scan showed metabolically active left axillary, bilateral supraclavicular, anterior mediastinal, subcarinal, and left internal mammary lymphadenopathy (Figure 3). Her screening breast mammography a few months prior to admission showed a benign‐appearing mass in the left breast and was read as BI‐RADS 2. On the present admission, a breast MRI was ordered, due to concern for a breast primary malignancy, and revealed multiple masses in both breasts that did not enhance and was read as BI‐RADS 2. However, given clinical suspicion for breast cancer, an ultrasound‐guided core biopsy of the mass was pursued and did not show tumor cells. Our patient's mother had breast cancer in her 40 seconds. She did not have a history of hormone replacement therapy or radiation treatment.

Of note, her screening breast mammography 2 years ago also exposed a benign‐appearing mass in the left breast and was read as BI‐RADS 2. A subsequent breast MRI at that time showed a 10 mm nodule**.** The MRI was also read as BI‐RADS 2, and no biopsy was pursued.

Excisional biopsy of the left axillary and left supraclavicular lymph node yielded poorly differentiated adenocarcinoma, with histopathologic features of ductal breast adenocarcinoma which was ER, PR, HER2 negative, and Ki‐67 positive.

She was started on abraxane and atezolizumab and is currently being followed in the outpatient oncology clinic.

## 3 DISCUSSION

As evidenced, by this case breast cancer can be difficult to diagnose despite advances in technology. Many modalities are available to aid in the diagnosis of breast cancer and include ultrasound, breast mammography, breast MRI, and biopsy. The sensitivities and specificities vary among these. Our patient had ultrasound, breast mammography, breast MRI, and ultrasound‐guided core biopsy of the left breast lump on her present admission, all of which did not reveal the underlying cancer.

According to the American College of Radiology, annual screening for breast cancer should begin at 40 years of age. There is no age limit, but each patient's life expectancy and comorbidities should be considered.^2^ Sensitivity for breast mammography in women of this age group is approximately 71%, largely due to denser breast tissue. Whereas sensitivity for 50‐69 years of age is >85%.^3^ The use of ultrasound as an adjunct to breast mammography for screening has shown an increase of sensitivity to 91% in women aged 40‐49 years.^4^ MRI as a screening modality for breast cancer has been limited to specific populations, however, sensitivity is around 99%.^5^ Newer modalities of breast imaging such as digital breast tomosynthesis (DBT) and contrast‐enhanced spectral mammography (CESM) have shown promise. DBT has shown possible risk‐benefit ratio for women aged 40‐49 years.^6^ CESM is highly sensitive and is a potential alternative to MRI.^7^

Our patient had a mammogram and breast MRI 2 years prior to her current presentation that demonstrated a left‐sided breast mass which was also read as BI‐RADS 2. It is likely that her disease started at that time and progressed until her current presentation at an advanced stage. Image findings consistent with BI‐RADS 2 are managed with routine mammographic screening.^8^ Her breast cancer may have been found at an earlier stage had she had repeat breast imaging. However, the time frame between these initial studies and the present admission was within an acceptable range for screening.

Our patient's background of SLE initially clouded the picture as pericardial effusions have been reported in SLE patients.^9^ Pericardial effusion in a patient with a known history of systemic lupus erythematous and a recent viral illness presents a diagnostic dilemma. Lupus involvement of the pericardium occurs frequently and is included under its diagnostic criteria.^9^, ^10^ Analysis of the effusion demonstrated a bloody effusion, and cytology was consistent with metastatic adenocarcinoma. Her effusion may have not otherwise been sampled had it not been large and causing symptoms. However, this procedure was performed due to suspicion for malignant involvement.

A review of multiple studies regarding systemic lupus erythematous and the development of malignancies shows a predilection toward non‐Hodgkin's lymphoma, lung, liver, vulvar, vaginal, and thyroid cancers. Curiously, lupus patients were noted to have a decreased risk of breast and prostate cancer. Specifically, it was noted that triple‐negative disease was less likely to occur, possibly due to impaired DNA repair via anti‐DNA antibody.^11^ Our patient's serology was anti‐DNA negative. Perhaps, there is an association between anti‐DNA and triple‐negative breast cancer that could be elucidated.

Metastatic involvement of the pericardium is rare. It can occur from either direct invasion by an adjacent primary tumor, or by lymphatic or hematogenous spread.^12^ The most commonly reported primary malignancies include bronchial carcinoma, breast cancer, leukemia, Hodgkin's disease, non‐Hodgkin's lymphoma, melanoma, gastrointestinal tumors, and sarcomas. Interestingly, a recent study in Denmark evidenced pericarditis as an indicator of certain occult malignancies, including breast cancer.^13^

With the presence of axillary and supraclavicular lymphadenopathy, we were able to make the diagnosis after excisional biopsy of the axillary lymph node. Her breast MRI 2 years prior was negative for any lymphadenopathy. A retrospective analysis of 5533 cases of first breast cancer, at a single cancer center, yielded just seven patients whose diagnosis was made from isolated axillary lymphadenopathy. The incidence measured in this study is similar to previous findings in the literature, with an estimated 0.1%‐0.8% of breast cancers being occult. Six of these patients underwent breast MRI prior to treatment with two having findings suggestive of cancer. Neither patient had biopsies of these lesions, and subsequent breast surgery showed benign pathology.^14^ Current guidelines suggest treating patients such as these if pathology and immunohistochemistry are consistent with breast cancer.^15^ CK7+/CK20− staining is found not only in breast but also in lung, endometrial, endocervical, pancreatic, and gastric adenocarcinomas. Breast adenocarcinoma can be further distinguished from the others based on GATA3 positivity.^16^

Unlike the previously mentioned cases, our patient had mammograms and a MRI with benign findings. She also had a breast biopsy of one of these lesions that did not show evidence of malignancy. A review of 988 needle breast biopsies found that 2.23% of the involved specimens were falsely negative.^17^ Extrapolating this to our patient, one can argue that either our case involved a false negative biopsy or perhaps there was a lesion not able to be seen on imaging.

## 4 CONCLUSION

Despite the advances in diagnostics, breast imaging modalities have limitations, and cases of breast cancer can go unnoticed. Women 40 years of age and above should be referred for breast cancer screening. Occult breast cancers comprise a small portion of breast cancers but should be recognized, nonetheless. Additionally, breast cancer should be considered in the workup of malignant pericardial effusions even in the absence of malignant breast masses on imaging.

## CONFLICT OF INTEREST

None declared.

## AUTHOR CONTRIBUTIONS

All authors contributed to this manuscript and approved of its final version.
